# In-vitro, in-vivo, and in-silico assessment of radical scavenging and cytotoxic activities of *Oliveria decumbens* essential oil and its main components

**DOI:** 10.1038/s41598-021-93535-8

**Published:** 2021-07-12

**Authors:** Tahereh Jamali, Gholamreza Kavoosi, Yousef Jamali, Saeed Mortezazadeh, Susan K. Ardestani

**Affiliations:** 1 Institute of Biochemistry and Biophysics, University of Tehran, Tehran, Iran; 2 Immunoregulation Research Center, Shahed University, Tehran, Iran; 3 Institute of Biotechnology, Shiraz University, Shiraz, Iran; 4 Biomathematics Laboratory, Department of Applied Mathematics, School of Mathematical Science, Tarbiat Modares University, Tehran, Iran; 5 Department of Biophysics, Tarbiat Modares University, Tehran, Iran

**Keywords:** Biochemistry, Cancer, Computational biology and bioinformatics, Drug discovery

## Abstract

We aimed to explore and compare new insights on the pharmacological potential of *Oliveria decumbence* essential oil (OEO) and its main components highlighting their antioxidant activity in-vitro, in-vivo, and in-silico and also cytotoxic effects of OEO against A549 lung cancer cells. At first, based on GC–MS analysis, thymol, carvacrol, *p*-cymene, and γ-terpinene were introduced as basic ingredients of OEO and their in-vitro antioxidant capacity was considered by standard methods. Collectively, OEO exhibited strong antioxidant properties even more than its components. In LPS-stimulated macrophages treated with OEO, the reduction of ROS (Reactive-oxygen-species) and NO (nitric-oxide) and down-regulation of iNOS (inducible nitric-oxide-synthase) and NOX (NADPH-oxidase) mRNA expression was observed and compared with that of OEO components. According to the results, OEO, thymol, and carvacrol exhibited the highest radical scavenging potency compared to *p*-cymene, and γ-terpinene. In-silico Molecular-Docking and Molecular-Dynamics simulation indicated that thymol and carvacrol but no *p*-cymene and γ-terpinene may establish coordinative bonds in iNOS active site and thereby inhibit iNOS. However, they did not show any evidence for NOX inhibition. In the following, MTT assay showed that OEO induces cytotoxicity in A549 cancer cells despite having a limited effect on L929 normal cells. Apoptotic death and its dependence on caspase-3 activity and Bax/Bcl2 ratio in OEO-treated cells were established by fluorescence microscopy, flow cytometry, colorimetric assay, and western blot analysis. Additionally, flow cytometry studies demonstrated increased levels of ROS in OEO-treated cells. Therefore, OEO, despite showing antioxidant properties, induces apoptosis in cancer cells by increasing ROS levels. Collectively, our results provided new insight into the usage of OEO and main components, thymol, and carvacrol, into the development of novel antioxidant and anti-cancer agents.

## Introduction

Biological oxidants can be formed as by-products of normal cellular metabolism like respiration and stimulation of macrophages. The superoxide-producing enzyme (NADPH oxidase, NOX) and nitric oxide producing enzyme (NOS) are the main contributors of reactive nitrogen/oxygen species production in animal cells especially at some pathological conditions^1^. These highly reactive radicals can oxidatively modify a variety of biomolecules, leading to cellular oxidative stress and death^2,3^. The cell death caused by these unstable reactive radicals and the change in the balance between oxidants and exogenous and endogenous antioxidants appear to be major contributors to aging and various diseases such as cancer^4,5^. Endogenous (enzymatic and non-enzymatic) antioxidants such as superoxide dismutase, peroxidase, catalase, and glutathione play a critical role in keeping optimal cellular functions and thus systemic health. However, under oxidative stress, endogenous antioxidants may not be sufficient, and dietary exogenous antioxidants such as vitamin C, vitamin E, carotenoids, and polyphenols may be required to maintain optimal cellular functions^6^.

In pathological conditions such as diabetes, cancer, autoimmune and cardiovascular diseases, reactive oxygen/nitrogen species production is overwhelmed on the endogenous antioxidant system, thus the human body needs natural antioxidants from edible plants and diet to keep free radicals at low levels^7^. Despite the progression of chemotherapy for cancer, due to multiple side effects and induction of drug resistance, leading to loss of efficiency, the need for safer drug compounds especially with the natural origin is felt. The medicinal plants and their phytochemicals have shown cytotoxic effects on cancer cell lines^8^. In these contexts, essential oils from medicinal plants are gaining popularity worldwide due to strong antioxidant, anti-inflammatory, and also anti-cancer effects^9^. *Oliveria decumbens* vent, a relatively less explored beneficial plant belongs to the Apiaceae family, is an endemic plant of flora Iranica, and grows in the hot climate of Iran's southwest. Traditionally, *O. decumbens* essential oil (OEO) is used to relieve thirst in children, diarrhea, indigestion, stomach and abdominal pain, fever, cold, and oxidative disease^10,11^. To our knowledge, there is little information about the effects of this essential oil in scientific sources. In recent years, a few in-vitro antioxidant studies have been performed on this compound^12,13^. However, its antibacterial and antifungal effects have been confirmed^14,15^. In addition, our previous study showed that OEO has a significant inhibitory effect on breast cancer cell lines in-vitro and in-vivo through promoting the apoptosis and immunomodulatory effects^16,17^. In this study, based on traditional applications and identification of monoterpenoid phenolic components in OEO reported in previous studies^10,18^, OEO was selected for the antioxidant and cytotoxic investigations. Here, the in-vitro radical scavenging activity of OEO was evaluated. Subsequently, the antioxidant effects of OEO in macrophage cells were studied and compared with that of its main components. Cellular studies aimed to investigate the modulating effects of these agents on the generation of ROS (reactive-oxygen-species) and NO (nitric-oxide) in macrophages. These oxidants derive from various enzymes, including NOX and iNOS, respectively. In the following, the levels of modulatory effects of OEO and its components on the expression of iNOS and NOX subunits (gp91^phox^, p67^phox^, p47^phox^, p40^phox^, and p22^phox^) mRNAs in LPS-induced macrophage cell lines were considered. Ultimately, to consider whether OEO has a direct effect on these enzymes and can inhibit their activity, OEO important phytochemicals, carvacrol, and thymol, were evaluated in-silico to perform the molecular docking for their interaction with iNOS and a part of p67^phox^–p47^phox^ complex. In the following, based on a high content of oxygenated monoterpenes components in *Oliveria*, the effect of OEO on the cytotoxicity of A549, a non-small lung cancer cell line, was studied, and eventually, the death mode induced by OEO was explored in cancer cells.

## Results and discussion

### Chemical composition

According to our GC/MS analysis, thymol (25.54%), carvacrol (23.12%), *p*-cymene (22.07%), and γ-terpinene (17.80%) were identified as the main components of OEO. Therefore, OEO mainly contained monoterpenoids (52.65%) and monoterpenes (47%) (Table 1). Some studies reported similar chemical compositions in *Oliveria* oil, however, there are important differences in the quality and quantity of these components^10,19^. The discrepancy in the chemical composition may be related to genetic and ornamental parameters as well as a seasonal collection of plant or preparation methods of essential oil.

**Table 1:** Chemical compositions of *Oliveria decumbens* essential oil (OEO) identified by gas chromatography and gas chromatography-mass spectrometry.

No.	RT	RI	formula	Compounds	Percent
1	5.31	925.65	C10H16	α-Thujene	0.36
2	5.49	932.66	C10H16	α-Pinene	0.31
3	5.90	948.20	C10H16	Camphene	0.06
4	6.53	972.32	C10H17	Sabinene	0.04
5	6.63	976.42	C10H16	β-Pinene	1.84
6	7.00	990.24	C10H16	Myrcene	0.59
7	7.45	1005.60	C10H16	α-Phellandrene	0.13
8	7.62	1010.52	C10H16	δ-3-Carene	0.07
9	7.83	1016.44	C10H16	α-Terpinene	0.59
10	8.16	1026.00	C10H14	p-Cymene	22.07
11	8.20	1027.02	C10H16	Limonene	2.34
12	8.27	1029.01	C10H16	β-Phellandrene	0.63
13	8.34	1030.89	C10H18O	1,8-Cineole	0.14
14	8.50	1035.67	C10H16	β-Ocimene	0.02
16	9.35	1059.67	C10H16	γ-Terpinene	17.80
17	10.34	1088.00	C10H16	Terpinolene	0.14
18	10.70	1098.12	C10H18O	Linalool	0.06
19	13.40	1165.10	C10H18O	Borneol	0.04
20	13.87	1176.62	C10H18O	Terpinen-4-ol	0.18
21	14.40	1189.71	C10H18O	α-Terpineol	0.08
22	16.24	1233.82	C11H16O	Thymol methyl ether	0.02
23	16.63	1242.99	C11H16O	Carvacrol methyl ether	0.02
24	18.59	1289.49	C10H14O	Thymol	25.54
25	18.89	1296.49	C10H14O	Carvacrol	23.12
26	21.30	1354.38	C12H16O2	Thymol acetate	0.01
27	22.13	1374.25	C15H24	α-Copaene	0.01
28	23.90	1417.39	C15H24	(E)-Caryophyllene	0.01
29	26.60	1484.34	C15H24	β-Selinene	0.02
30	28.00	1519.83	C11H11O_3_	Myristicin	3.43
31	30.15	1575.50	C15H24O	Spathulenol	0.03
32	30.33	1580.11	C15H24O	Caryophyllene oxide	0.01
				Monoterpenes	46.992
				Monoterpenoids	52.646
				Sesquiterpenes	0.04
				Sesquiterpenoids	0.045

Retention indices were determined using retention times of n-alkanes as standard on fused silica capillary HP-5 column that was injected after essential oil under the same chromatographic conditions.

### Extracellular antioxidant capacity

In-vitro non-biological (ABTS and DPPH) and biological radicals (superoxide ion and nitric oxide) scavenging activity of OEO in comparison with standard antioxidant gallic acid were summarized in Table 2. Based on In-vitro analysis, OEO emulsion displayed a concentration-dependent ABTS, DPPH, superoxide anion, and NO scavenging activities (with IC_50_ of 36.7, 53.0, 89.0, and 101.7 µg/ml respectively) somewhat similar to gallic acid activity (with IC_50_ of 26.6,39.0, 91.3, and 109.3 µg/ml respectively). Additionally, thymol displayed the scavenging activities with IC_50_ of 61.6, 98.3, 151.6, 140.6 µg/ml respectively and carvacrol displayed that with IC_50_ of 67.0, 96.0, 141.6, and 167.3 µg/ml respectively. Therefore, thymol and carvacrol exhibited a radical scavenging activity with a similar capacity of apocynin, while *p*-cymene and γ-terpinene did not show any such radical scavenging. The potency of radical elimination, when ordered, was OEO > apocynin > thymol > carvacrol > cymene = terpinene > L-NAME. The high antioxidant capacity of OEO emulsion against biological and non-biological oxidants may be linked to the strong synergism between oxygenated monoterpenes or phenolic monoterpenes in the cocktail of essential oil^20^. The antioxidant properties of phenolic compounds are attributed to their redox potentials^21,22^, which permit them to be strong hydrogen donors, radical oxygen quenchers, and metal-chelating agents. Phenolic compounds, through the donation of the hydrogen from hydroxyl groups to free radicals, prevent the oxidation of other compounds^23^. Essential oils scavenge reactive oxygen/nitrogen species, inhibit lipid oxidation and therefore reduce damage in the biological cell membrane and protect tissues and cells against oxidative damage^24,25^.

**Table 2:** The antioxidant capacity of OEO, main components, apocynin, L-Name (positive controls in in-vivo studies) comparison with standard antioxidant gallic acid.

Antioxidants	ABTS	DPPH	Superoxide ion	Nitric oxide
Gallic acid (µg/ml)	26.6 ± 1.12^a^	39.0 ± 1.25^a^	91.3 ± 2.92^a^	109.3 ± 3.50^a^
Apocynin (µg/ml)	59.7 ± 2.09^c^	88.6 ± 3.10^c^	123.7 ± 4.33^b^	157.3 ± 5.50^c^
L-NAME (µg/ml)	900 ± 12.60^e^	900 ± 10.0^e^	900 ± 11.40^e^	900 ± 12.60^e^
OEO (µg/ml)	36.7 ± 1.14^b^	53.0 ± 1.64^b^	89.0 ± 2.76^a^	101.7 ± 3.15^a^
Thymol (µg/ml)	61.6 ± 2.09^c^	98.3 ± 3.34^c^	151.6 ± 5.15^c^	140.6 ± 4.78^b^
Carvacrol (µg/ml)	67.0 ± 2.21^c^	96.0 ± 3.17^c^	141.6 ± 4.67^c^	167.3 ± 5.52^c^
Cymene (µg/ml)	500 ± 11.00^d^	500 ± 10.00^d^	500 ± 12.00^d^	500 ± 10.00^d^
Terpinene (µg/ml)	500 ± 12.50^d^	500 ± 10.50^b^	500 ± 11.50^d^	500 ± 10.50^d^

The concentrations of OEO that could provide 50% radical or oxidant inhibition (IC_50_) were calculated from the graph that plotted the radical or the oxidant inhibition percentage against different antioxidant concentrations. The values are expressed as means ± SDs for three replicate experiments. Mean values with different letters within a column are significantly different by the Tukey test at (*p* < 0.05).

### MTT assay

For antioxidant studies on macrophage cells, we need to prepare the appropriate concentrations of the OEO and its components without the lethal effect on the cells. Therefore, an MTT assay was performed. According to the results, OEO and its components had no effects on the macrophage cell viability at low concentrations (5 and 10 μg/ml). However, at high concentrations, all of them induced cytotoxicity by 50%. Therefore, the concentrations of 5 and 10 μg/ml of components were selected for the following studies. Indeed, these are concentrations that we can easily use for antioxidant studies on macrophage cells.

### Intracellular ROS scavenging activity and NOX down-regulation

**Figure 1:**
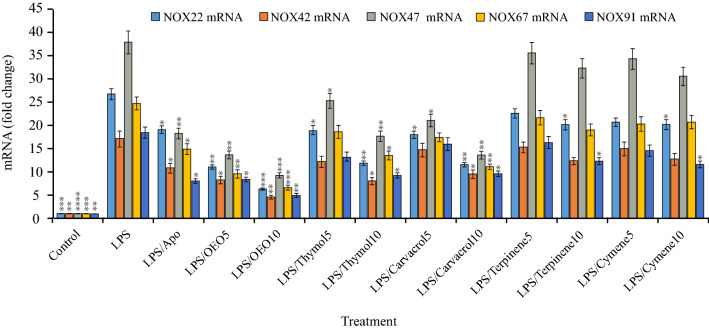
Effects of OEO, main components, and positive control (apocynin) on the NOX mRNA expression in LPS-stimulated macrophages. Mean values are significantly different by Tukey test at *p < 0.05, **p < 0.01, ***p < 0.001 and ****p < 0.0001 versus LPS group.

Macrophage cells were incubated or not in the presence of 2 µg/ml LPS together with non-cytotoxic concentrations (5 and 10 µg/ml) of OEO, thymol, carvacrol, *p*-cymene, γ-terpinene, and apo (10 μg/ml). Our analysis showed that the stimulation of macrophages by LPS induces ROS generation. Treatment with OEO, carvacrol, and thymol significantly reduced ROS in LPS-treated cells (Table 3). The stimulation of macrophages by LPS led to increasing the rate of NOX mRNA expression and activity. A marked decrease in mRNAs expression and activity of NOX was detected in LPS-stimulated cells treated with OEO emulsion, thymol, and carvacrol (Fig. 1). However, γ-terpinene and *p*-cymene caused no significant change in the level of NOX activity and mRNAs expression.

**Table 3:** Effects of OEO, main components, and positive control (apocynin) at different concentrations (5 and 10 µg/ml) on ROS production and NOX activity in LPS-stimulated macrophages.

Treatment	ROS(× 1000)	NOX activity
Control	15.7 ± 0.86^b^	11.7 ± 0.64^a^
LPS	27.6 ± 1.32^ef^	32.0 ± 1.54^f^
LPS/Apo10	17.6 ± 0.77^bc^	13.3 ± 0.59^ab^
LPS/OEO5	14.3 ± 0.72^b^	22.3 ± 1.12^d^
LPS/OEO10	9.7 ± 0.57^a^	13.7 ± 0.81^b^
LPS/Thymol5	20.3 ± 1.14^c^	20.3 ± 1.14^d^
LPS/Thymol10	16.0 ± 0.82^b^	14.3 ± 0.73^bc^
LPS/Carvacrol5	17.3 ± 0.85^b^	16.0 ± 0.78^c^
LPS/Carvacrol10	10.3 ± 0.57^a^	10.0 ± 0.55^a^
LPS/Cymene5	25.0 ± 1.40^de^	25.6 ± 1.43^e^
LPS/Cymene10	24.6 ± 1.30^cd^	22.6 ± 1.20^d^
LPS/Terpinene5	29.9 ± 1.50^f^	25.7 ± 1.29^e^
LPS/Terpinene10	25.0 ± 1.43^d^	22.7 ± 1.29^de^

The values are expressed as means ± SDs for three replicate experiments. Mean values with different letters within a column are significantly different by the Tukey test at (*p* < 0.05). This test classifies the groups into letters. Groups that have little significant difference have similar letters and those with more significant difference receive different letters.

Based on our data, OEO exhibited comparable ROS scavenging activity and NOX down-regulation more than its main components however, carvacrol and thymol had a little more inhibitory effect on NOX activity. The better performance of the OEO than other chemical components can be attributed to the multi-component nature of OEO and the synergy between the monoterpenes, monoterpenoids, and phenolic monoterpenoid components. According to previous studies, several plants containing monoterpenes and monoterpenoids have been identified to display reducing activity in ROS production, NOX expression, and activity in LPS-stimulated cells at the non-cytotoxic level^26–28^.

In macrophages, superoxide production via NOX is the main mechanism. NOX, as a multi-protein enzyme consists of various subunits (gp91phox, gp67phox, gp40phox, gp47phox, and gp91phox). The active form of this enzyme is produced by the assembly of subunits in the plasma membrane. LPS-activated NOX converts molecular oxygen to superoxide^29^. After binding to toll-like receptor 4 (TLR-4) and activating the gp91phox subunit, LPS leads to the production of superoxide. Superoxide or hydrogen peroxide radical (from superoxide dismutase activity) activates MAPK families. MAPKs eventually stimulate the production of interleukin-6 (IL-6), IL-22, IL-8, IL-17, cyclooxygenase-2, and iNOS by activating nuclear factor-κB (NF-κβ). Accordingly, ROS itself can induce NO^30^. The underlying mechanisms of essential oils in inhibiting the production of superoxide are still unknown. These inhibitory effects may be directly related to the decreased NOX expression, the inhibition of TLR-4, or the downstream members of the LPS signaling pathway such as NF-κB or others that need further investigation. Based on our study, OEO decreased the expression of the main subunits of NOX and also NOX activity. Therefore, we can confirm that an important part of the reduction of NOX activity induced by OEO is due to the decreasing expression of NOX subunits and the low assembly of these subunits on the membrane. The suppression of NOX expression and activity indicated the ability of OEO to decrease superoxide production and provide more evidence for OEO anti-oxidative properties.

### Intracellular NO scavenging and NOS down-regulation

**Figure 2:**
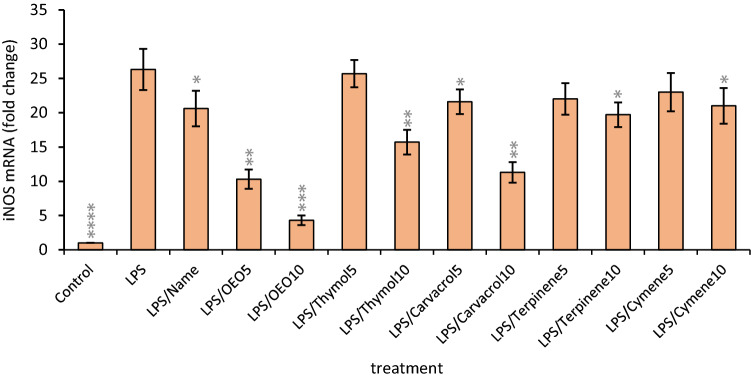
Effects of OEO, main components, and positive control (L-NAME) on NOS mRNA expression in LPS-stimulated macrophages. Mean values within a column are significantly different by Tukey test at *p < 0.05, **p < 0.01, ***p < 0.001 and ****p < 0.0001 versus LPS group.

Macrophage cells were incubated or not in the presence of 2 µg/ml LPS together with non-cytotoxic concentrations (5 and 10 µg/ml) of OEO, thymol, carvacrol, *p*-cymene, γ-terpinene, and L-NAME (1.3 µg/ml). LPS stimulation of macrophages increased NO level while treatment with OEO, carvacrol, and thymol significantly decreased NO production respectively, indicating the inhibitory effect of these molecules on NO generation (Table 4). LPS stimulation increased iNOS mRNA expression while OEO, carvacrol, and thymol reduced iNOS expression and activity in LPS-treated cells. However, *p*-cymene and γ-terpinene caused little change in the level of iNOS mRNAs expression and activity (Fig. 2). The results are means ± SDs (*P* < 0.05). Collectively, this data showed that OEO exhibits strong antioxidant properties even more than its main components however, carvacrol and thymol showed a little more inhibitory effect on iNOS activity. The better activity of OEO can be related to the multi-component nature of OEO and a combination of additive and/or synergistic effects of various components. The inhibitory effects of monoterpenoids and monoterpenes bearing essential oil from *Houttuynia cordata*^31^, *Rimulus cinnamon*^32^, and *Daphne oleoides Schreb*^33^ on NO production and NOS expression/activity in LPS-stimulated macrophages and neutrophil have been confirmed.

**Table 4:** Effects of OEO, main components at different concentrations (5 and 10 µg/ml), and positive control (L-NAME, 1.3 μg/ml) on NO production and NOS activity in LPS-stimulated macrophages.

Treatment	NO	NOS activity
Control	27.0 ± 1.22^de^	13.3 ± 0.60^a^
LPS	38.0 ± 2.13^g^	42.3 ± 2.37^f^
LPS/L-Name1.3	21.0 ± 1.22^c^	17.3 ± 1.00^b^
LPS/OEO5	21.3 ± 1.26^c^	31.7 ± 1.87^cd^
LPS/OEO10	14.7 ± 0.84^a^	17.6 ± 1.00^b^
LPS/Thymol5	25.6 ± 1.41^d^	28.3 ± 1.56^c^
LPS/Thymol10	18.0 ± 1.04^b^	17.3 ± 1.00^b^
LPS/Carvacrol5	21.7 ± 1.17^d^	19.0 ± 1.03^b^
LPS/Carvacrol10	12.0 ± 0.70^a^	11.3 ± 0.66^a^
LPS/Cymene5	29.0 ± 1.51^e^	38.7 ± 2.01^ef^
LPS/Cymene10	24.7 ± 1.33^d^	32.7 ± 1.77^d^
LPS/Terpinene5	32.7 ± 1.87^f^	36.7 ± 2.13^e^
LPS/Terpinene10	28.0 ± 1.43^e^	33.6 ± 1.71^de^

The values are expressed as means ± SDs for three replicate experiments. Mean values with different letters within a column are significantly different by the Tukey test at (*p* < 0.05). This test classifies the treated groups into letters. Groups that have little significant difference have similar letters and those with more significant difference receive different letters.

NO in macrophages is generated through oxidation of l-Arg by iNOS. NOS can produce high levels of NO after stimulation with pro-inflammatory cytokines or bacterial endotoxins. Inflammatory stimuli such as LPS stimulate the macrophages to produce various inflammatory mediators (such as TNF-α). The TNF-α is a crucial stimulus for the induction of NO synthesis^34^. LPS binds to TLR-4 and activates MAPK like; P38 MAPK, ERK, and also JNK. MAPKs finally activate NF-κB that itself stimulates iNOS expression and NO production^35^. But, the real mechanism of essential oil on the inhibition of NO generation in stimulated macrophages is unknown. These inhibitory effects may be directly related to the reduction of NOs expression/activity or the inhibition of TLR-4 receptor or other downstream elements in NO signaling like MAPK, NF-κB, or other transcription factors that more researches are needed to confirm. Our results showed that OEO significantly reduces NOS mRNA expression and activity and finally NO production in stimulated macrophages. Therefore, we can confirm that an important part of the reduction of NOS activity induced by OEO is because of the decreasing the NOS expression. The reduction of NOS gene expression because of OEO shows the ability of this agent to diminish oxidative stress.

Altogether, in-vivo results perfectly indicated the ability of OEO to diminish the oxidative reactions even better than thymol and carvacrol which may be related to the strong synergism between the components in the OEO cocktail.

### Molecular modeling of ligands–NOX interaction

**Figure 3:**
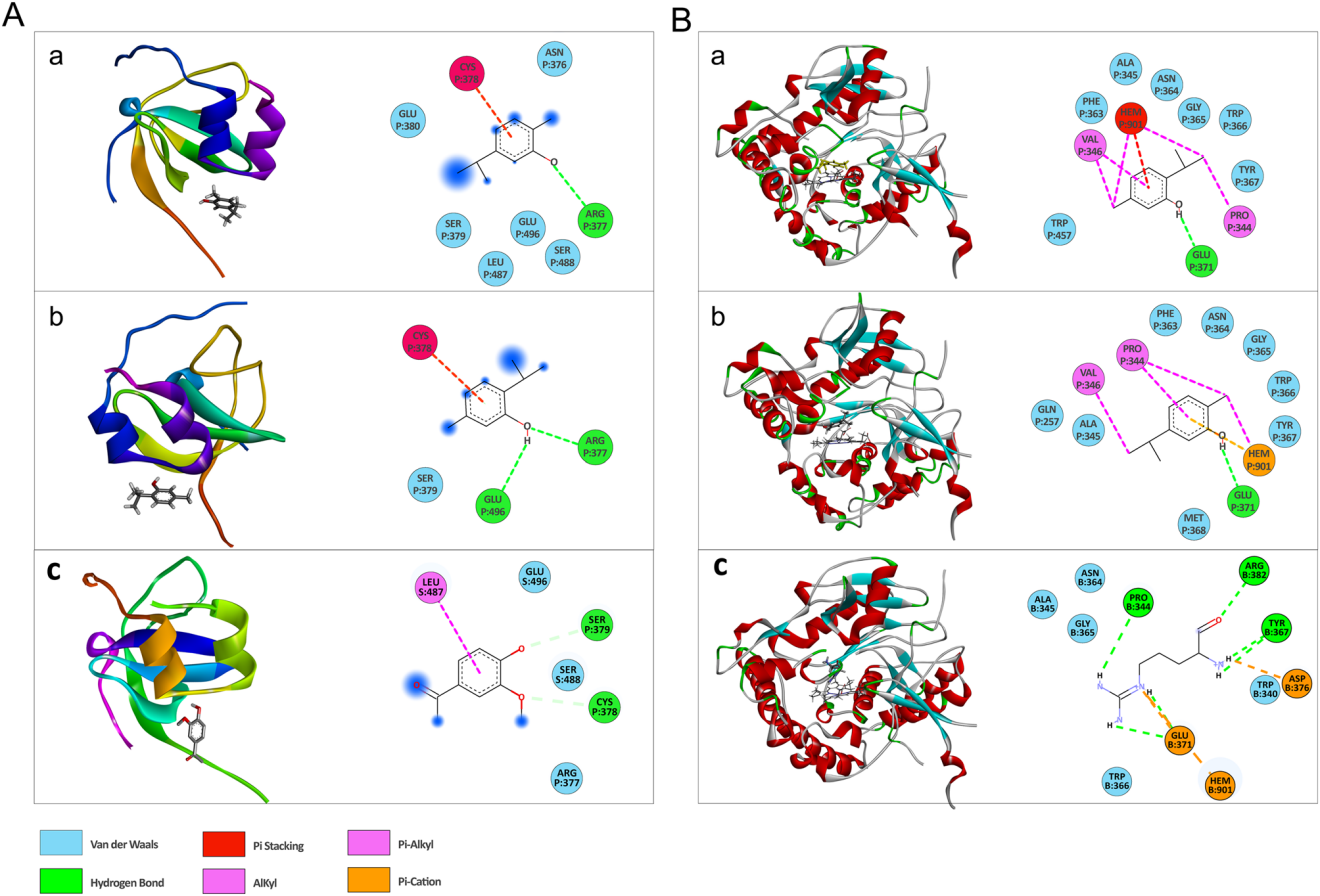
The interactions (**A**) between NOX (PDB ID:1K4U) and carvacrol (a), thymol (b), apocynin (c) (**B**) between iNOS (PDB ID:1nod) and carvacrol (a), thymol (b), and l-Arg (c). Conventional hydrogen bonds have been labeled using green dashed lines.

Based on our in-vitro results and to explore whether the main components of OEO have any interaction with a subsection of NADPH oxidase (NOX, PDB: 1K4U), docking of thymol, carvacrol, and apocynin (as a positive control) with 1K4U was performed. 1K4U subsection consists of the C-terminal SH3 domain of p67phox complexed with the C-terminal tail region of p47phox. Since thymol/carvacrol has some structural similarities with apocynin (an inhibitor of NADPH oxidase), the active site (with the key residue cysteine-378) was selected based on that identified in published papers in the interaction of apocynin (apo) with 1K4U^36^. The docking results showed the involvement of the phenyl ring of thymol/carvacrol into the pocket near CYS378 of 1K4U and Pi interaction between ligand and receptor. Additionally, H-bond and van der Waals interaction between thymol/carvacrol and 1K4U were revealed (Fig. 3). The binding energies for docking of carvacrol/thymol to 1K4U were found to be − 4.4 and − 4.3 kcal/mol respectively. The binding energy value of carvacrol/thymol and comparing it to that of apocynin (− 6.5 kcal/mol) showed that carvacrol/thymol interacts with 1K4U subsection with less affinity than apocynin and therefore exhibited a weaker inhibitory effect on NADPH oxidase. According to the docking results, γ-terpinene/*p*-cymene exhibited no interaction with 1K4U and confirmed that they have no inhibitory effect on NADPH oxidase. Therefore, the inhibitory effect of OEO and its main components may probably not be due to binding to and occupation of the active site of the NOX enzyme.

### Molecular docking of ligands–iNOS interaction

In macrophages, NO biosynthesis is carried out from l-Arg and is catalyzed by iNOS. l-Arg, as a substrate, binds to glutamic acid-371 in the catalytic center of iNOS and stacks with the heme group in a hydrophobic pocket^37^. In this study, the interaction of the main components of OEO and l-Arg with 1nod (murine cytokine-inducible nitric oxide synthase oxygenase dimer) was investigated by the docking method. The results showed that carvacrol and thymol are docked almost at the l-Arg binding site with the binding energy of − 20 and − 18 kcal/mol respectively in comparison to that of l-Arg (− 22 kcal/mol). These ligands interact with GLU-371 in the catalytic center with hydrogen bonds and have interactions with the heme group. Therefore, H-bond, Pi interactions, and van der Waals interactions between thymol/carvacrol and 1nod active site were revealed which provided proper affinity between ligands and receptor (Fig. 3). However, both *p*-cymene/γ-terpinene docked almost at an active site with weak binding energy (− 6.3) through Pi and van der Waals interactions. Therefore, our results suggest that thymol/carvacrol may be able to inhibit iNOS availability for l-Arg by blocking the position of l-Arg. Therefore, the decrease in iNOS activity by OEO, containing large amounts of thymol and carvacrol, may be due to the blockage of the active site and a decrease in enzyme activity.

### Molecular dynamic (MD) simulation

**Figure 4:**
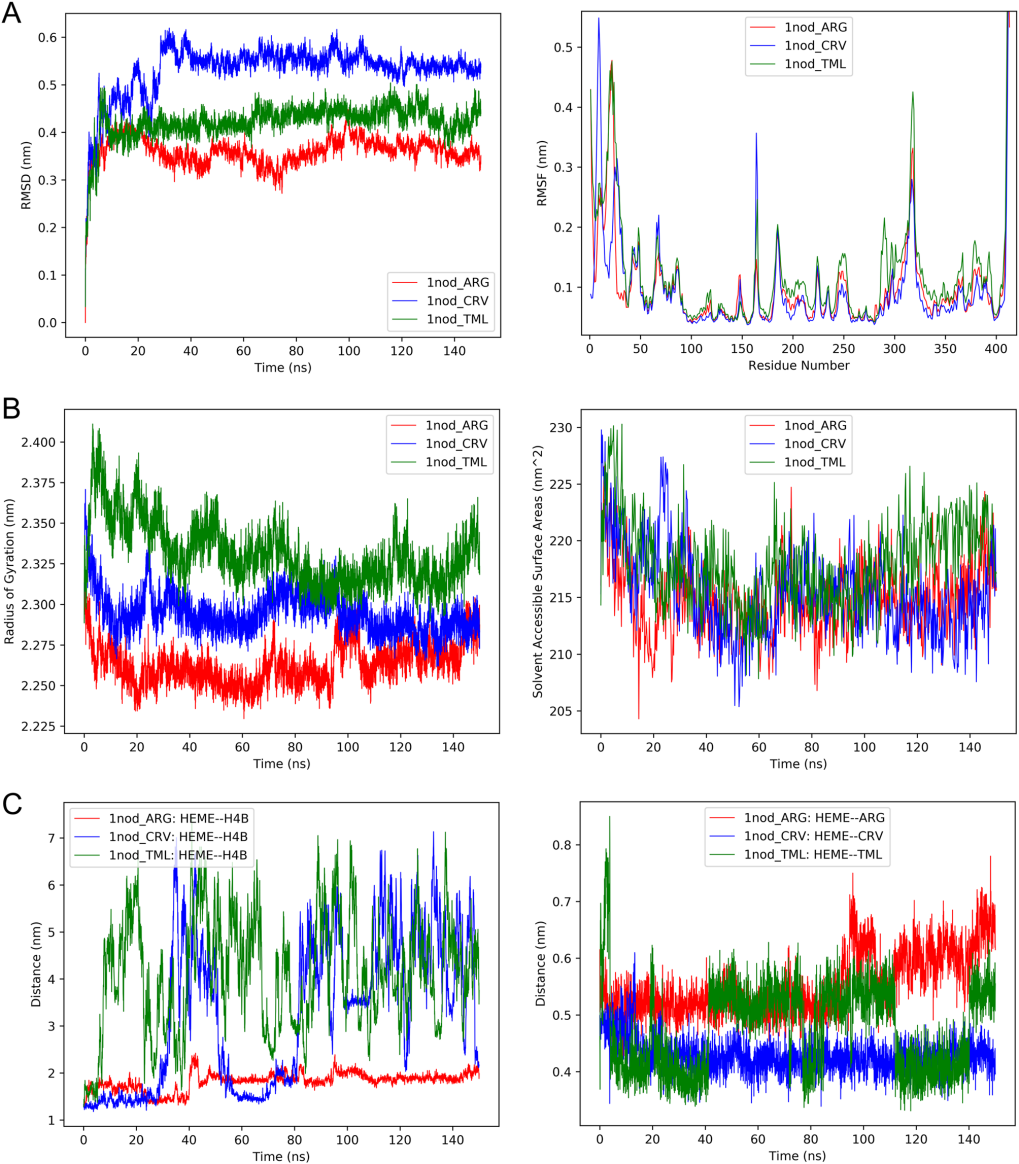
(**A**) Right: the RMSD and left: RMSF of C_α_ of iNOS protein during the simulation. (**B**) Right: the radius of gyration and left: solvent accessible surface area of iNOS in case of bonding to the native ligand and the drugs. (**C**) Right: distances between the center of mass of the heme group and the enzyme’s cofactor tetrahydrobiopterin (H4B) and left: distances of the heme group with the Arg and drug molecules during the simulation.

The root mean square deviation (RMSD) and root mean square fluctuation (RMSF) of the C_α_ atoms of iNOS protein during the simulation are depicted in Fig. 4A. For the correct comparison of RMSD values, the same reference was selected for all three modes. The first thing to get from the RMSD values is that all three systems are in good thermodynamic equilibrium so that RMSF values were calculated after 50 ns.

Second, the protein structure distance when binding to thymol and carvacrol is significant compared to the natural Arg ligand. This indicates considerable changes in protein structure due to agent binding. After binding these agents, the structural fluctuations of the protein, especially around the active site, increase dramatically (Fig. 4A). Increased protein flexibility leads to an increase in the radius of gyration which indicates a partial opening in the protein structure (Fig. 4B). This increase in protein radius was more pronounced when the thymol molecule binds to the protein, which is consistent with the RMSF results. Also, the solvent-accessible surface area (SASA) of the protein is higher when the thymol is attached than in the other two cases (Fig. 4B). The average of SASA values after 50 ns of simulation are as follows: 214.734 ± 2.805 (nm^2^) for 1nod_ARG, 214.137 ± 2.959 (nm^2^) for 1nod_CRV, and 217.458 ± 3.542 (nm^2^) for 1nod-TML. The opening of the protein structure in the presence of thymol causes it to be exposed to aqueous solvents more than the other two substances.

The binding of thymol and carvacrol to the iNOS protein causes the H4B cofactor to be cleaved from the enzyme (Fig. 4C), which inactivates the enzyme. However, these agents remained stable at their binding sites during the MD simulation (Fig. 4C). Since the thymol molecule can form hydrogen bonds to Trp366 and Met368, in some cases its distance from the heme group is similar to that of the heme with Arg.

The native substrate of iNOS (Arg) can bind to the protein more strongly than thymol and carvacrol (Table 5).

**Table 5:** Free energy binding of arginine, thymol, and carvacrol to the iNOS enzyme.

	Van der Waals energy (kJ/mol)	Electrostatic energy (kJ/mol)	Polar solvation energy (kJ/mol)	SASA energy (kJ/mol)	Binding energy (kJ/mol)
1nod-ARG	− 61.02 ± 14.59	− 625.11 ± 33.03	517.79 ± 36.66	− 11.44 ± 0.65	− 179.78 ± 25.73
1nod-CRV	− 129.18 ± 6.62	− 5.66 ± 5.82	52.89 ± 10.14	− 11.11 ± 0.52	− 93.06 ± 9.62
1nod-TML	− 104.42 ± 7.13	− 14.85 ± 3.69	52.79 ± 5.9	− 11.21 ± 0.61	− 77.69 ± 7.93

This is due to the ability to form strong hydrogen and electrostatic bonds between Arg and the enzyme. Therefore, the contribution of electrostatic interactions to the binding of Arg to protein is greater than that of van der Waals energy. On the contrary, van der Waals interactions play a major role in binding the thymol and carvacrol to the protein. Together, the results of MD were consistent with the above results and broadly confirmed the possibility of binding of thymol and carvacrol to the active site of iNOS and subsequent inactivation of the enzyme.

Altogether, in-silico results supported that the inhibitory effect of OEO on NOX activity is through reduced mRNA expression of enzyme subunits while that of on iNOS may be due to both reduced iNOS mRNA expression and the blockage of enzyme active site by OEO major components, thymol, and carvacrol.

### OEO cytotoxicity in A549 cells

The cytotoxicity of OEO on A549 along with L929 cells was evaluated after 24 h using MTT assay. The lower cytotoxicity of OEO was observed on L929 cells when compared with A549 cells. OEO exhibited a dose-dependent decline in the growth of A549 cells. IC_50_ values of OEO were found to be 22.14 and > 100 μg/ml in A549 and L929, respectively. Therefore, OEO in proper concentration can inhibit the growth of cancer cells. It is clear that the lower the effective concentration of a drug, the better drug will be and the fewer side effects it will show. In addition, these results confirmed that despite the significant cytotoxic effect of OEO on A549 cells, OEO does not indicate any growth-inhibitory effect on L929 normal cells. Moreover, the effect of Etoposide as a positive control on A549 cells was considered and IC_50_ has calculated about 6.5 μg/ml (Table 6). The results are the means ± SDs from triplex experiments (*P* < 0.05).

**Table 6:** Cell inhibition percentage after treatment with OEO and Etoposide for 24 h.

Cell line	Components	Components concentration (µg/ml)							
		5	10	15	20	30	40	80	IC_50_
A549	OEO	4 ± 2.3	28 ± 4.04	38 ± 2.3	50 ± 1.15	56 ± 1.15	63 ± 1.7	89 ± 3.2	22.14
L929	OEO	0	3 ± 1.7	3 ± 1.7	5 ± 0.5	11 ± 1.2	13 ± 1.2	16.5 ± 0.7	> 100
A549	Etoposide	32.6 ± 1.5	54 ± 3.0	67 ± 1.15	75 ± 1.7	82 ± 2	92 ± 2.5	93 ± 2.5	6.5

Growth inhibition percentage and IC_50_ (the concentration of OEO or Etoposide at which 50% of cell proliferation are inhibited) were determined using the MTT assay. The results are the means ± SDs from triplex experiments by the Tukey test at (*p* < 0.05).

Therefore, OEO possesses an appropriate selectivity between cancer and normal cells. In previous reports, we exhibited the anti-cancer effects of OEO and thymol on the human MDA-MB231 cell line^16^. Anti-cancer activity of carvacrol on several cancer cell lines was confirmed^38–40^. OEO is a hydrophobic liquid that easily passes through the membrane and possibly causes cell death through various signaling pathways. According to the anti-cancer studies of phenolic components, the anti-cancer effects of OEO may be reflected in the synergism between monoterpenes and monoterpenoids in the cocktail of OEO. Essential oils due to their lipophilic nature can enter into cytoplasmic membranes increasing the fluidity of the membrane. Displacement of essential oils between the fatty acid chain of phospholipids and glycolipids enhances fluidity and permeability of membranes resulting in leakage of cations like calcium. The calcium leakage triggers cells to apoptosis and necrosis^41^. Alternatively, phenolic components can be oxidized by reactive nitrogen/oxygen species, mitochondrial electron transport chain, or some heavy metals. These reactions lead to the production of additional radical species such as phenoxyl radicals. These radicals are able to oxidize DNA, proteins, and lipids and therefore cause necrosis and/or late apoptosis. If the essential oil concentrations are not high enough to make the mitochondria permeable, their conversion into pro-oxidant do not occur, and eventually the antioxidants keep its activity. In contrast, at high concentrations, they could damage the mitochondria. Essential oils also can be oxidized into pro-oxidants and act as the pro-oxidants and consequently led to cell death.

### Induction of apoptosis in A549 cells

**Figure 5:**
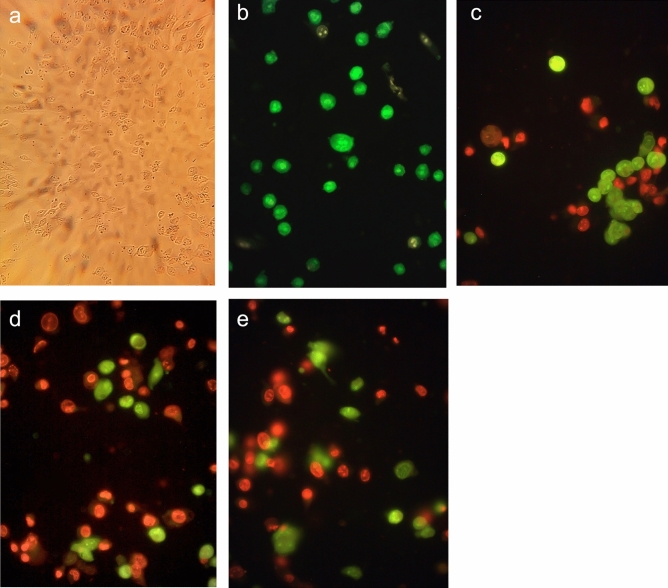
(**a**) Treated A549 cells with OEO (IC_50_) under an inverted microscope. (**b**) Control A549 cells and (**c**) treated cells with OEO (1/2IC_50_) for 24 h. (**d**) Treated cells with OEO (IC_50_) for 24 h. (**e**) Treated cells with Etoposide (positive control) (IC_50_) for 24 h. The cells were stained using AO and EtBr and visualized with fluorescence microscopy.

Observation of A549 cells in the presence of OEO after 24 h using an inverted microscope showed noticeable morphological changes. Some sequential changes are cell shrinkage and rounding, detachment, and cytoplasmic vacuolation (Fig. 5a). These changes strongly suggested that OEO may induce apoptosis in A549 cells. Therefore, several studies were performed to determine whether OEO-induced death was apoptotic.

In AO/EB fluorescence staining, the differentiation between cells is based on the differential uptake of dyes in cells. Live cells are stained with AO and appear green while cells in the early apoptotic stage stained with both AO and EB, are green/orange and have condensed chromatin. Late apoptotic cells are orang with fragmented and condensed chromatin and are stained with EB. Fluorescence microscopic examination of A549 cells treated with OEO (IC_50_ and ½ IC_50_) and Etoposide (IC_50_) (Fig. 5) confirmed the late apoptosis in the cells treated with OEO/Etoposide after 24 h.

#### AnnexinV/PI staining

**Figure 6:**
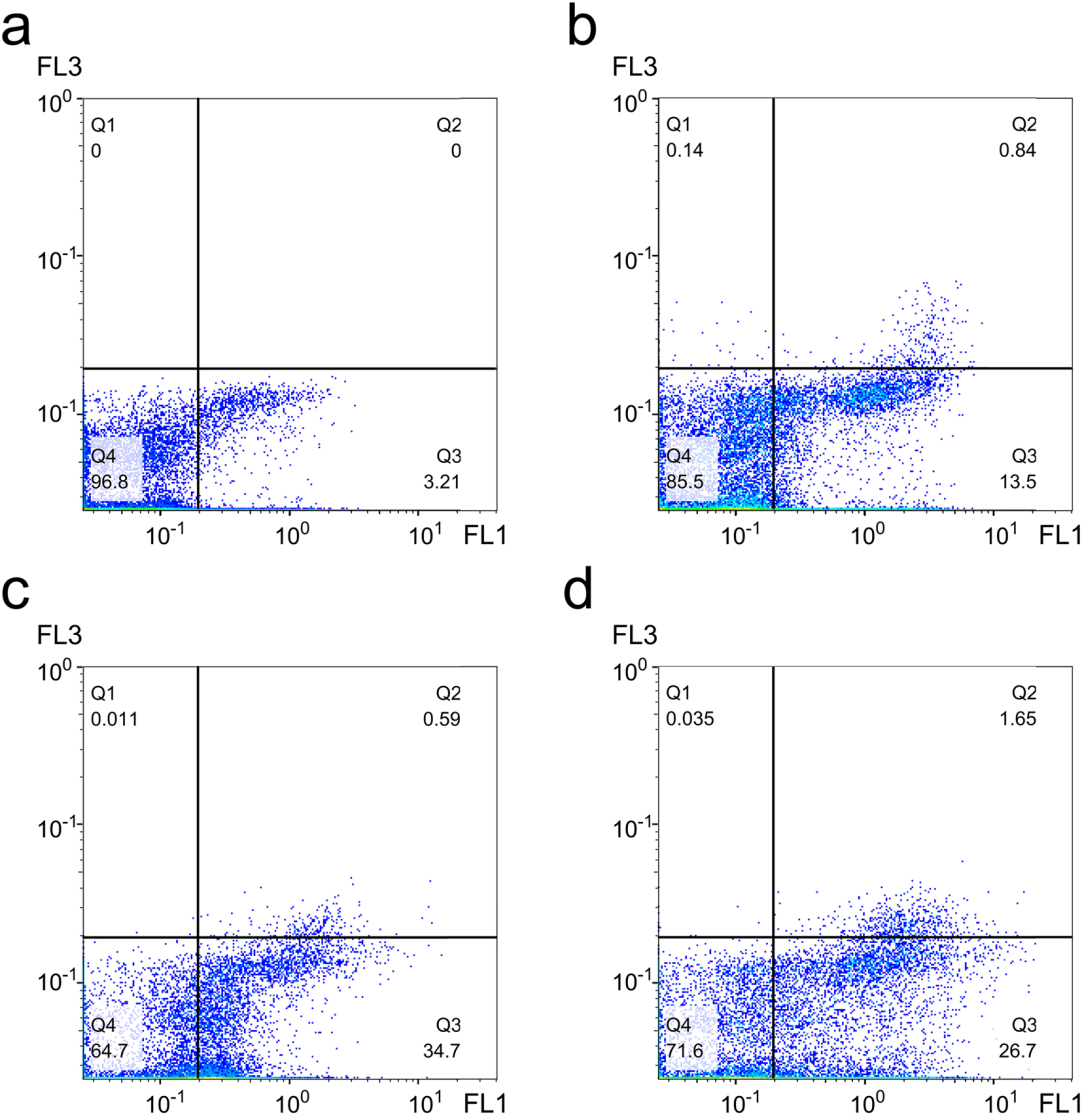
AnnexinV-FITC/PI analysis by flow cytometry in (**a**) control cells and (**b**) treated cells with OEO (1/2 IC_50_) for 24 h (**c**) Treated cells with OEO (IC_50_) for 24 h (**d**) Treated cells with Etoposide (positive control) (IC_50_) for 24 h. Alive cells are Annexin V-FITC and PI negative; cells in early apoptosis are Annexin V-FITC positive and PI negative (lower right quadrant), and cells in late apoptosis or necrosis are both Annexin V-FITC and PI-positive (upper right quadrant).

An event during apoptosis is the flipping of phosphatidylserine (PS) to the outer surface of the cell membrane. AnnexinV, a calcium-binding protein, binds to PS and thus FITC-labeled AnnexinV can spot PS in apoptotic cells. Therefore, exposure to PS can be detected using AnnexinV in the ruptured plasma membrane of necrotic and apoptotic cells. Besides, the co-staining of cells with PI provides a way to distinguish the necrotic from apoptotic cells. Flow cytometry-based detection with AnnexinV-FITC and PI in treated A549 cells can quantify apoptosis in treated cells. The percentage of apoptotic cells measured by flow cytometry compared to control cells showed that after 4 h treatment, OEO in ½ IC_50_ and IC_50_ value and Etoposide in IC_50_ induces early apoptosis (FITC-Annexin-V+/PI−) in A549 cells about 4.2, 10.8, and 8.3 fold rather than control cells respectively. Thus, the inhibitory mode induced by OEO in A549 cells is apoptosis (Fig. 6).

### Induction of apoptosis by OEO via a caspase-3 dependent pathway

Caspase-3 is the main effector caspase and cleaves an extensive spectrum of the substrates caused apoptosis. Investigation of p-NA absorbance (obtained from cleavage of caspase substrate) in the treated and control samples determined the fold increase in caspase-3 activity. In this study, the detection of caspase-3 activity exhibited that caspase-3 activity increased in treated cells with OEO (½ IC_50_ and IC_50_) about 3 and 4.6 respectively comparisons to control cells. Caspases play an essential role in apoptosis. Mitochondrial membrane permeability in the apoptotic process leads to the release of cytochrome C from mitochondria to cytosol and the activation of caspase cascades and apoptosis. Here, our data from the consideration of caspase 3 activity confirmed that OEO can trigger apoptosis through a caspase-3 dependent pathway.

### Modulation of Bax/Bcl-2 ratio by OEO

**Figure 7:**
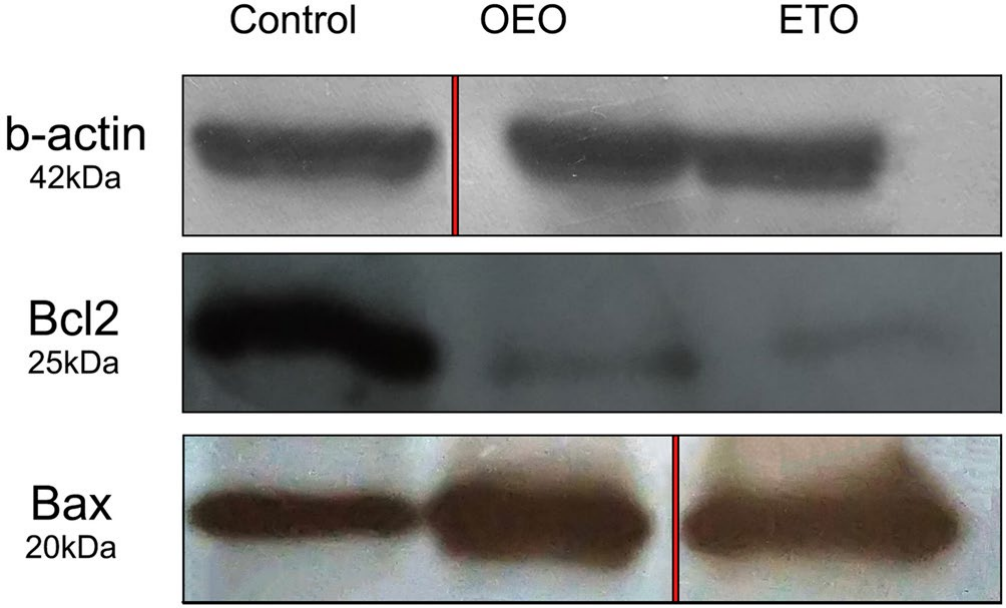
Western blot analysis of Bcl-2 (anti-apoptotic protein) and Bax (pro-apoptotic protein) expression in control cells and treated cells with OEO (IC_50_) and Etoposide (positive control) (IC_50_) for 24 h. The raw figures of blots has been provided in the “Supplementary information”

As noted above, the apoptosis induced by OEO is associated with caspase-3 activity. In the following, in this article, to consider the apoptotic mechanisms, the effect of different treatments on the expression of Bcl-2 (anti-apoptotic) and Bax (pro-apoptotic) proteins were also studied. Since Bcl-2 family members act as the important regulators of mitochondrial outer membrane permeability and an increase in the ratio of Bax/Bcl-2 is connected to mitochondrial permeability, this relation was discovered by a study of Bax and Bcl-2 expression in OEO-treated cells. The expression level of Bcl2 was found to decrease in the treated cells (with IC_50_ of OEO for 24 h) compared to the control cells while the expression level of Bax was observed to upregulate (Fig. 7). Therefore, since increasing the level of the Bax leads to mitochondrial membrane damage, resulting in cytochrome C release and caspase-9 activation, followed by activation of caspase-3, our result confirmed that OEO induces apoptosis through modulation of Bax/Bcl-2 ratio and ultimately triggers mitochondrial apoptosis pathway with promoting the downstream signaling pathways to the death.

### Induction of apoptosis via intracellular ROS generation by OEO

**Figure 8:**
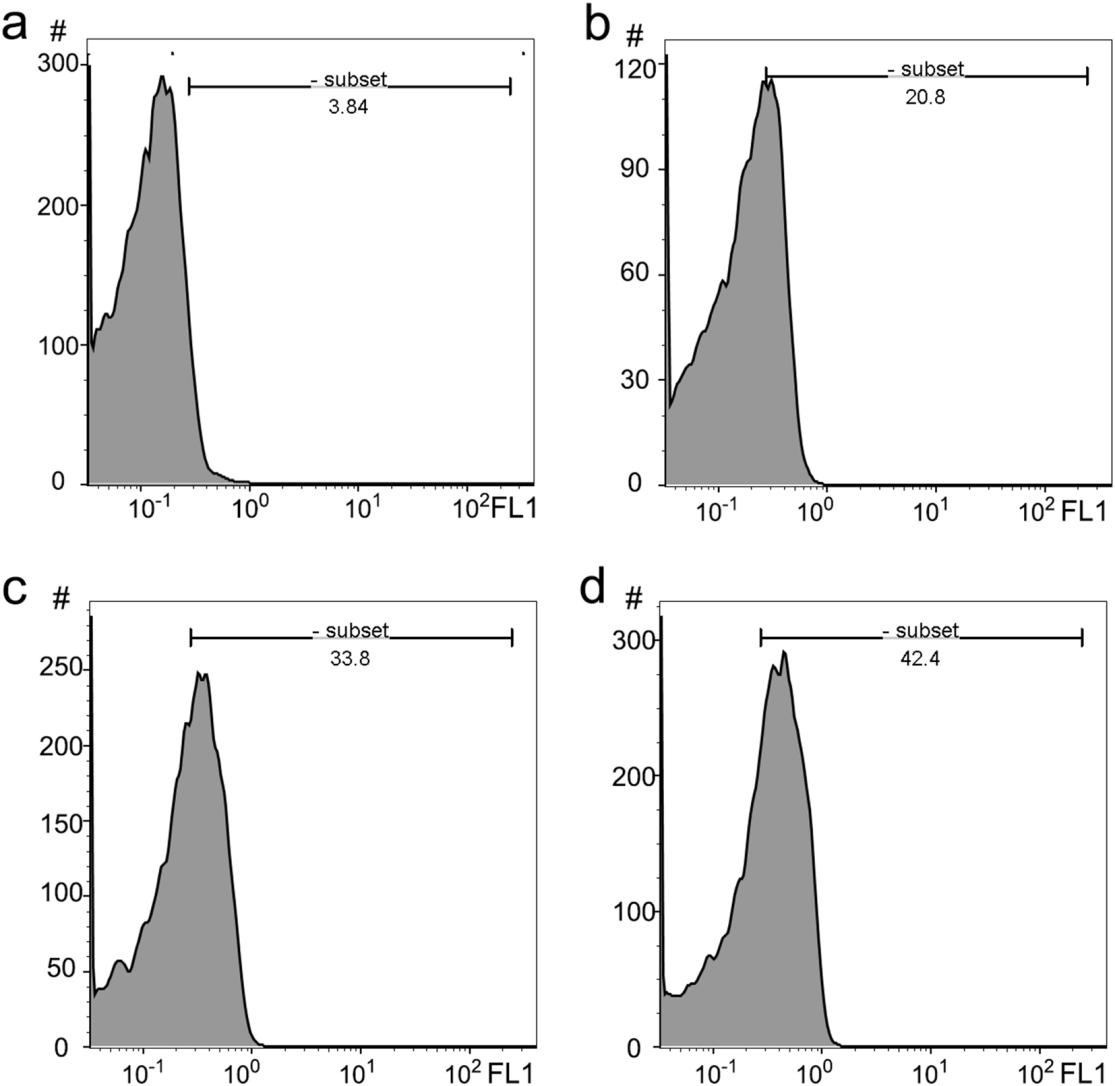
ROS analysis by flow cytometry in (**a**) A549 control cells and (**b**) A549 treated cells with OEO (1/2 IC_50_) for 24 h (**c**) A549 treated cells with OEO (IC_50_) for 24 h d) A549 treated cells with the Etoposide (positive control) (IC_50_). The x-axis shows log FL-1 fluorescence intensity; the y-axis indicates the cell number. The shift of the population to the right in treated cells compared to control cells indicates the apoptotic cell population.

Cancer cells usually show a high level of ROS. This may cause the progression of the disease or may make cancer cells more susceptible to extra ROS. Accumulation of ROS plays an important role in cytotoxicity induced by anticancer agents and the regulation of apoptosis. Some plants and phytochemicals develop antioxidant defenses in cancer cells while other plants and derived compounds enhance the ROS level leading to cytotoxicity^42^. However, the biological mechanisms involving the ROS level in apoptosis are various and not precisely clear. ROS-induced oxidative stress directly or through increasing the membrane permeability, cytochrome C discharge, DNA damage, and cell cycle arrest can promote apoptosis. Here, to determine whether ROS production or antioxidant activity is responsible for the anti-proliferative effects of OEO, the treated A549 cells were monitored by DCFH-DA subjected to flow cytometry. Our studies showed that OEO in ½ IC_50_ and IC_50_ significantly induces oxidative stress and elevates the levels of ROS about 8.80 and 5.41 fold respectively in treated A549 (Fig. 8). Therefore, based on our results, although OEO exhibits strong antioxidant properties in different conditions, it can trigger ROS-mediated cytotoxicity in A549 cancer cells. Indeed, the biochemical pathways triggered by plant phytochemicals are complex, and different studies are needed to understand this complexity.

## Materials and methods

This study was carried out in compliance with the ARRIVE guidelines and all methods including animal methods were carried out in accordance with relevant guidelines and regulations. The relevant ethical guidelines were followed for the experiments involving plants.

### Preparation and chemical composition of OEO

*Oliveria decumbens* (with the local name of “moshkorak” and “denak”) is known as the only species of the genus Oliveria (http://www.ipni.org/ and https://www.gbif.org). The aerial parts of this plant (herbarium number: 55078) were collected from the mountainous areas of Fars, in southwestern Iran in the spring of 2016. The identification of collected plants was thankfully done by Professor Ahmad Reza Khosravi, plant taxonomist at Biology Department, Shiraz University, Iran. The permissions were not necessary for the collection of the plant. However, we only collected as many plants as needed in our research.

OEO was extracted from the air-dried plants through hydrodistillation for 3 h using an all-glass Clevenger-type apparatus (Herbal Exir Co., Mashhad, Iran). Indeed, 70–100 g plant materials were soaked in 1000–1200 ml water and placed in a glass balloon, and heated for 3 h at 100 °C. Steam from boiled water contains the most volatile chemicals. The steam was then cooled and the distillate was collected. OEO floated on top of the aromatic water and separated through a separating funnel. The yield of OEO was 2.5% (w/w). In the following, OEO was dehydrated over anhydrous sodium sulfate and then was stored at 4 °C. The average OEO density was gained by a digital balance (Acculab, Sartorius Group, Germany) and informed about 1000 mg/ml^16,17^.

GC/MS of OEO was carried out using an Agilent 7890 A series gas chromatograph with a flame ionization detector (FID) (Agilent, Palo Alto, CA, USA). The analysis was performed on a fused silica capillary HP-5 column of 30 m × 0.32 mm i.d. and film thickness of 0.25 μm. Helium was the carrier gas at a flow rate with a split ratio of 1/40. The injector volume was 0.1 μl and the temperature program was 60–240 °C. Injector and detector temperature were kept at 240 °C and 250 °C respectively. GC–MS was fitted with an Agilent gas chromatography coupled with an Agilent model 5975 C mass spectrometer equipped with a column HP-5MS. The temperature and carrier gas (helium) was similar to that of the above. The temperature of the Ionization source was maintained at 280 °C. OEO elements were identified by comparison of Retention indices (based on the homologous series of n-alkanes (C8–C25)) with those informed in the literature and the internal reference libraries (Wiley GC/MS Library and Mass Finder 2.1 Library)^43^.

### Preparation of OEO and main components emulsion

To prepare the essential oil emulsion, 1.0 ml of OEO (equivalent to 1 g) was added to 100 ml of distilled water or aromatic water obtained as a byproduct in the process of hydrodistillation. In this study, we used distilled water. Essential oils are not soluble in water. Therefore, we need an emulsifier. In this study, we used polysorbate-20 (a polysorbate-type nonionic surfactant) as an emulsifier. Polysorbate-20 (100 µg/ml) was added to OEO-water and the mixture was maintained at 35 °C for 24 h. At this time, a milky emulsion was formed. In the presence of an emulsifier, essential oil micelles were prepared and solubilized in water. OEO emulsion was adjusted to 1000 µg/ml of gallic acid equivalent using deionized water for antioxidant characterization^44^. To perform extracellular tests, the diluted emulsion was redissolved in distilled water, and to perform cell tests, the emulsion was diluted in the culture medium to obtain the desired concentrations (in the antioxidant study: 5 and 10 μg/ml and in the anticancer study: 0–100 μg/ml). Main components of OEO including thymol (CAS 89-83-8), carvacrol (CAS 499-75-2), p-cymene (CAS 99-87-6), and γ-terpinene (CAS 99-85-4) were purchased from Sigma-Aldrich. In addition to these components, L-Name (CAS 51298-62-5), apocynin (CAS 498-02-2), and gallic acid (CAS 149-91-7) were also purchased for antioxidant studies. These components were solved in 1% DMSO (dimethyl sulfoxide) to obtain a stock solution. In the following, all the agents were dissolved in water (in-vitro studies) or culture medium (in-vivo and in-vivo studies) to obtain a working solution.

### ABTS/DPPH radical scavenging assay

OEO (0–200 µg/ml) or gallic acid (0–200 µg/ml) was added to dilute ABTS radical solution (1 ml) or DPPH solution (0.2 mM in 95% methanol). After shacking the obtained mixture and incubating for 30 min (at room temperature, in the dark), the absorbance was measured at 734 nm for ABTS and 517 nm for DPPH. The percentage of ABTS/DPPH radical elimination = [(A_A/D_ − A_t_)/A_A_] × 100. A_A/D_ = absorbance of ABTS/DPPH solution without a sample (OEO) and A_t_ = absorbance of the ABTS/DPPH solution mixed with the sample. Ultimately, IC_50_ (the required concentrations that could inhibit 50% ABTS/DPPH radical) was measured^45^.

### Superoxide or nitric oxide radical scavenging assay

OEO (0–200 µg/ml) was incubated with a superoxide reaction mixture (1.0 ml) for 15 min (at ambient temperature). The reaction mixture in phosphate buffer (10 mM, pH 7.4) contains phenazine methosulfate (20 mM), nicotinamide adenine dinucleotide (300 mM), nitro blue tetrazolium (50 mM). After incubation, the absorbance was recorded at 560 nm^46,47^. For NO scavenging assay, OEO (0–200 µg/ml) was incubated with sodium nitroprusside (SNP) (0.5 ml from 20 µg/ml prepared in 100 mM sodium citrate pH 5) at 37 °C. After 2 h incubation, a Griess reagent (0.5 ml) (1:1 of 0.1% naphthylethylenediamine and 1% sulfanilamide in 5% phosphoric acid) was added and then the absorbance of the solution was recorded at 540 nm^48^.

The percentage of superoxide/NO radical elimination = [(A_control_ − A_t_)/A_control_] × 100. A_control_ = absorbance of superoxide/NO reaction solution and A_t_ = absorbance of superoxide/NO in the presence of OEO. Ultimately, a graph plotted with the percentage of superoxide/NO inhibition against different OEO concentrations was used to determine IC_50_.

### Experimental animals

Forty BALB/c mice (male, 10–12 weeks old) were purchased from the Pasteur Institute of Iran (Tehran, Iran). Mice were housed under filter top conditions with enough water and food. They were used for experimental purposes with the approval of the Animal Ethics Committee of the Ministry of Health and Medical Education (IR.TMU.REC.1394.193) (Tehran, Iran). Macrophage cells were removed from the peritoneal fluid and then the treatments were performed on the cells. It should be noted that the mice used, after collecting macrophages, were delivered to another group of researchers for further studies.

### Cell harvest and culture

Macrophage cells were harvested from peritoneal fluid of BALB/c mice in sterile PBS (5.0 ml, pH 7.4). First, the outer skin of the peritoneum was cut so that the inner skin covering the peritoneal cavity appears. Using a 27 g needle, gently 5 ml of cold PBS was injected into the peritoneal cavity. After the injection, the peritoneum was gently massaged. Then, with a 25 g needle attached to a 5 ml syringe, the liquid was collected from the peritoneum. These procedures were repeated several times and as much fluid as possible was collected. Finally, the collected cell suspension was placed in a tube and on ice. The cells were washed two times with PBS and centrifuged (at 1700×*g* for 5 min). The cells were then cultured in RPMI-1640 medium (Gibco, Carlsbad, CA). RPMI-1640 was supplemented with l-glutamine (2 mM), antibiotics (100 µg/ml streptomycin and 100 U/ml penicillin,) and 10% FCS (Gibco, Carlsbad, CA). Cell viability was checked visually by trypan blue dye exclusion. Cultures were maintained in a humidified CO_2_ incubator at 37 °C. The cells were grown in culture plates in triplicate. After 20 h, non-adherent cells were discarded by gentle washing with warm RPMI-1640, and adherent cells were cultured^49^.

### Cytotoxicity of OEO, thymol, carvacrol, p-cymene, and γ-terpinene

Cytotoxicity was quantified by MTT assay. Peritoneal exudate murine macrophage cells (harvested from 2 mice) were adjusted to a density of 1.4 × 10^4^ cells/well respectively and seeded in 96 well plates and plates were incubated for 24 h. After this, macrophage cells were treated with different concentrations of OEO, thymol, carvacrol, *p*-cymene, and γ-terpinene (0–200 µg/ml) then were maintained in 5% CO_2_ for 24 h at 37 °C. Next, the supernatant was removed and 200 μl/well of MTT solution (0.5 mg/ml in PBS) was added to each well. After 4 h incubation at 37 °C in dark, the solution was removed and DMSO was added (100 μl/well). With incubation on a shaker, formazan crystals were dissolved. Finally, the absorbance was detected using a microplate reader (BioTek, Winooski VT, USA) at 492 nm^50^.

### Determination of intracellular NO in macrophages

Macrophage cells (harvested from 15 mice) were seeded into a 24 well tissue culture plate (2 × 10^6^ cells/ml, 1 ml/well) and incubated for 24 h (at 37 °C under humidified air with 5% CO_2_) to adhere. Macrophage cells were incubated or not in the presence of 2 µg/ml LPS (2 µg/ml, from *Escherichia coli*) together with non-cytotoxic concentrations of OEO, thymol, carvacrol, *p*-cymene, γ-terpinene or positive control, L-NAME (1.3 µg/ml). After incubation for 24 h, culture supernatant (100 µl) were mixed with Griess reagent (100 µl) (1:1 of 0.1% naphthylethylenediamine and 1% sulfanilamide in 5% phosphoric acid) in 96 well plates, incubated at room temperature (for 10 min) and the nitrate content was determined at 540 nm in a microplate reader (BioTek, Winooski VT, USA). Sodium nitrite at different concentrations was applied for standard curve fitting to estimate nitrate values via extrapolation^51,52^.

### Determination of intracellular reactive oxygen species (ROS) in macrophages

Intracellular ROS was measured using 2′,7′-dichlorodihydrofluorescin diacetate (DCFH2-DA). DCFH2-DA is capable of diffusing through membranes and is hydrolyzed by intracellular esterase to get the free acid DCFH2. This molecule rapidly oxidized to the highly fluorescent 2′,7′-dichlorofluorescein (DCF). Indeed, the fluorescence intensity is relative to the hydrogen peroxide level produced in the cells. Cells (harvested from 2 mice) were added to 96-well plates (2 × 10^4^ cell/well, 200 µl/well) and incubated for 24 h. The culturing medium was removed and replaced with a fresh one. Macrophage cells were incubated or not in the presence of 2 µg/ml LPS together with non-cytotoxic concentrations (5 and 10 µg/ml) of OEO, thymol, carvacrol, *p*-cymene, γ-terpinene or apocynin (10 µg/ml) for 20 h. Then DCFH2-DA (2 µg/ml) was added to the medium and then incubated for an additional 2 h in the dark. Finally, fluorescence was monitored at 485 nm (an excitation wavelength) and 528 nm (an emission wavelength) using a fluorescent microplate reader^53^.

### NADH oxidase and nitric oxide synthase activity

Cells (harvested from 15 mice, 2 × 10^6^ cells/ml) were washed with culture medium and lysed with 1% SDS in water. 0.5 ml of cell lysate was added to a vial containing 0.5 ml of sodium phosphate buffer (100 mM, pH 7.5) containing protease inhibitors (protease inhibitor mix, 80-6501-23, Healthcare, Germany). NOX activity was determined at 340 nm using potassium phosphate buffer (100 mM, pH 7.5) containing NADH (100 µM) and dithiotreitol (1 mM). The reaction was initiated after adding 0.5 ml of cell homogenate to 0.5 ml of the above solution (Hummel & Riebel, 2003). NOS activity was determined using a NOS assay kit (Calbiochem, 482702, UK). The value was expressed as a unit per ml.

### Quantitative RT-PCR

Macrophages cells (harvested from 2 mice) were added to 96-well plates (2 × 10^4^ cell/well, 200 µl/well) and incubated for 24 h. Macrophage cells were incubated or not in the presence of 2 µg/ml LPS together with non-cytotoxic concentrations (5 and 10 µg/ml) of OEO, thymol, carvacrol, *p*-cymene, γ-terpinene, L-NAME (1.3 µg/ml) and apo (10 μg/ml) for 20 h. RNA was extracted using the RNX-plus buffer (Cinagen, Tehran, Iran). Macrophage cells were transferred to RNX-plus buffer (1 ml) in an RNase-free microtube and left for 5 min at room temperature. Then the chloroform (0.2 ml) was gently added to the mixture. The mixture was centrifuged at 12,000*g* for 10 min at 4 °C. An equal volume of isopropanol was added to the resulting supernatant and incubated for 15 min on ice to appear the RNA pellet. Thereafter, the RNA was washed with ethanol (75%), dried, and resuspended in RNase-free water (0.015 ml). The quality of RAN was assessed by agarose gel electrophoresis and measuring light absorbance at 260 nm by nanodrop ND1000 spectrophotometer (Nano-Drop, Wilmington, DE) (260/280 absorbance ratio of ~ 2.0). After performing RNA nanodrop, DNase treatment was performed according to Fermentas DNase Kit (Fermentase, Hanover, MD). Total RNA (1 mg), DNase buffer (10×, 2 ml), DNase enzyme (1 U/ml, 2 ml), and DEPC water (15 ml) were mixed, reached a 100 ml total volume with DEPC water, and placed at 37 °C for 30 min. Then the quantity and quality of RNA were measured again by nanodrop and 1% agarose gel electrophoresis. The extracted samples were then kept at − 80 °C. The first-strand cDNA synthesis was initiated from RNA (1 µg), oligo-dT (100 pmol), dNTPs (15 pmol), RNase inhibitor (20 U), and M-Mulv reverse transcriptase (200 U) in a 0.02 ml final volume using first-strand cDNA kit (Fermentas, Hanover, MD). The synthesized cDNA was then used as a template in quantitative real-time PCR. Primers (in form of exon junction) were designed using Allele ID 7 software for the internal control glyceraldehydes-3-phosphate dehydrogenase (GAPDH) (NM-008084) and examination genes p22phox (cytochrome b-245, alpha polypeptide (Cyba), NM-007806.3), p40phox (neutrophil cytosolic factor 4 (Ncf4), NM-008677.2), p47phox (neutrophil cytosolic factor 1 (Ncf1), NM-001286037.1), p67phox (neutrophil cytosolic factor 2 (Ncf2), NM-010877.4), gp91phox (cytochrome b-245, beta polypeptide (Cybb), NM-007807.5) and inducible nitric oxide synthase (iNOS, NM-010927) genes (Table 7).

**Table 7:** Designed primers used in qPCR, melting temperature (Tm) of the primers, and the size of their specific products.

Genes	Accession no.	Primer	Sequence	Tm	Product length
GAPDH	NM-008084	Sense sequence	5^′^-CGGTGTGAACGGATTTGGC-3^′^	58	139
GAPDH	NM-008084	Anti-sense sequence	5^′^-TGAGTGGAGTCATACTGGAAC-3^′^	58	139
iNOS	NM-010927	Sense sequence	5′-CTGGAGGTTCTGGATGAG-3′	58	179
iNOS	NM-010927	Anti-sense sequence	5′-CTGAGGGCTGACACAAGG-3′	58	179
NOX p22	NM-007806.3	Sense sequence	5′-ATGGAGCGATGTGGACAG-3′	62	104
NOX p22	NM-007806.3	Anti-sense sequence	5′-ACCGACAACAGGAAGTGG-3′	62	104
NOX p40	NM-008677.2	Sense sequence	5′-CAACAAAGACTGGCTGGAG-3′	54	204
NOX p40	NM-008677.2	Anti-sense sequence	5′-CCGCAATGTCCTTGATGG-3′	54	204
NOX p47	NM-001286037.1	Sense sequence	5′-ATGGCACAAAGGACAATC-3′	60	157
NOX p47	NM-001286037.1	Anti-sense sequence	5′-ACCTGAGGCTATACACAAG-3′	60	157
NOX p67	NM-010877.4	Sense sequence	5′-CAGCCACAGTCAGCAGAG-3′	52	189
NOX p67	NM-010877.4	Anti-sense sequence	5′-GCACAAAGCCAAACAATACG-3′	52	189
NOX p91	NM-007807.5	Sense sequence	5′-TGT GGCTGTGATAAGCAGGAG TTC-3′	59	115
NOX p91	NM-007807.5	Anti-sense sequence	5′-TTGAGAATGGAGGCAAAGGGCG-3′	59	115

The relative standard curve method was used to analyze and quantify the relative gene expression. Subsequently, the data were normalized to the absolute control group and also to the gene expression of GAPDH. The amplification reactions were performed in a line-Gene K thermal cycler (Bioer Technology Co., Hangzhou, China). For quantitative RT-PCR data, relative expressions of NOX and iNOS genes were done based on the threshold cycle (CT) method. To calculate the CT value for each sample, Line-gene K software was used. Accordingly, the expression level of target mRNAs was calculated using the 2^−ΔΔCT^. ΔCT was determined by subtraction of the corresponding internal control CT value from the specific CT of targets, and ΔΔCT was obtained by subtraction of the ΔCT of each experimental sample from that of the control sample^54^.

### Molecular modeling

The 3D structures of OEO main components were downloaded from http://www.chemspider.com, and iNOS and NADPH oxidase structures were downloaded from Protein Data Bank (PDB ID: 1nod and 1K4U). The molecular docking was accomplished by AutoDockTools-1.5.6/Vina from The Scripps Research Institute (http://autodock.scripps.edu/references). The ligands and receptor files were changed into PDBQT format, polar hydrogen atoms were added and the water molecules were removed. The grid parameters were selected as follows: for 1nod binding site (center_x = 125.446, center_y = 115.958, center_z = 93.644, size_x = 46, size_y = 44, size_z = 46) and for 1K4U binding site (center_x = 5.831, center_y = −2.718, center_z = 7.158, size_x = 30, size_y = 24, size_z = 12). Finally, all figures were visualized using PYMOL software and the docking scores as binding free energy (ΔG) were reported.

Molecular dynamics simulation is one of the important tools for investigating the structural stability of proteins in the vicinity of drugs. Gromacs 2019.3^55^ was used to simulate nitric oxide synthase (iNOS) grafted to three different ligands including Arg, carvacrol, and thymol. In this study, the CHARMM 36^56^ force field for the protein molecule and the TIP3P^57^ model for the water molecule was used. The CHARMM General Force Field (CGenFF) version 2.2.0^58^ was also used to parameterize the molecules of carvacrol, thymol, and 5,6,7,8-tetrahydrobiopterin. The systems containing a protein with the docked ligands were dissolved in a cubic box with water molecules and the net charge of the systems was neutralized by adding the NA ions. Then, the steepest descent method was used to minimize the system’s potential energy up to 10,000 steps. The time step was set to 2 fs alongside using the P-LINCS^59^ algorithm to constrain the motion of hydrogen atoms. The MD simulations were carried out in the NPT ensemble for 150 ns at temperature 300 K by using V-rescale thermostat^60^ with the time constant 0.5 ps and pressure 1 bar by using and Parrinello–Rahman barostat^61^ with the time constant 5 ps. The fast smooth Particle-Mesh Ewald (PME)^61^ method with a cutoff radius of 1.2 nm was adjusted to calculate electrostatic interactions. The g_mmpbsa software^62^ was used to calculate the binding energy of three different Arg, carvacrol, and thymol ligands to the protein. Also, the Ligplot + software^63^ was used to generate of 2D ligand–protein interaction diagrams.

### Cell culture of A549 and L929

A549 (non-small cell lung cancer) along with L929 (normal fibroblast cell line) were purchased from Pasteur Institute, Iran. These cells were cultured in RPMI-1640 medium (Gibco, Carlsbad, CA) containing FBS (10%) (Gibco, Carlsbad, CA), antibiotics (100 μg/ml streptomycin, 100 U/ml penicillin) (Cinagen, Tehran, Iran). Cells were maintained at the incubator (37 °C under humidified air containing 5% CO_2_) and were harvested using 0.25% trypsin/EDTA (Gibco, Carlsbad, CA)^64^.

### Cytotoxicity assay

MTT assay was used to investigate cell viability. A549 and L929 cell lines (1 × 10^4^ cells/well) were grown in 96-well plates and were treated with various concentrations of OEO (0–100 µg/ml) for 24 h. In this study, A549 cells were also treated with Etoposide (as a positive control, dissolved in 1% DMSO and diluted with culture medium, (0–100 μg/ml)). Next, the supernatant was removed and 200 μl/well of MTT solution (0.5 mg/ml in PBS) was added to each well. After 4 h incubation at 37 °C in dark, the solution was removed and DMSO was added (100 μl/well). With incubation on a shaker, formazan crystals were dissolved. Finally, the Absorbance was detected using a microplate reader (BioTek, Winooski VT, USA) at 492 nm^65,66^. Subsequently, IC_50_ (the half-maximal (50%) inhibitory concentration of cell proliferation) was estimated from the concentration–response curves^17^.

### Detection of apoptosis in A549 cell lines

#### Fluorescent staining

A549 cells were seeded at a final concentration of 2 × 10^5^ cells/ml in 6-well culture plates and were treated with IC_50_ of OEO or Etoposide for 24 h. Then cells were washed once with PBS and were plated onto glass slides. Ethidium Bromide (EB)/Acridine Orange (AO) containing solution (100 mg/ml) (Sigma-Aldrich, Steinheim, Germany) was added and the cells were immediately pictured with a fluorescence microscope (Axioskop 2 plus, Zeiss, Germany)^67^.

#### AnnexinV-FITC and PI staining

To identify the apoptotic and necrotic A549 cells treated with OEO or Etoposide, the AnnexinV-FITC apoptosis kit (BioVision, Milpitas, CA, USA) was used. The 3 × 10^6^ A549 cells were plated in 6-well plates containing 3 ml of complete medium and incubated for 24 h. After treatment for 4 h with IC_50_ and ½ IC_50_ concentrations of OEO and also IC_50_ of Etoposide, the cells were harvested by 0.25% trypsin/EDTA, washed using ice-cold PBS, and stained with PI and FITC according to the protocol of the kit manufacturer. The cells were incubated for 15 min (at ambient temperature, in dark). Flow cytometry (Partec PAS, Munich, Germany) was used to identify the percentage of labeled cells^16^.

### Caspase-3 activity assay

Caspase-3 activity was assayed using a caspase-3 colorimetric activity assay kit (BIOMOL International, PL, USA). This study is based on a spectrophotometric assay of p-nitroaniline (p-NA) once the cleavage of caspase-3 substrate, Ac-DEVD-pNA (Sigma, St. Louis, MO, USA). About 3 × 10^6^ A549 cells were exposed to OEO (½ IC_50_ and IC_50_) and Etoposide (IC_50_) for 4 h. After harvesting the cells and washing with cold PBS, the cells were lysed by cell lysis buffer [PIPES (20 mM), Mgcl (2 mM), KCl (10 mM), DTT (4 mM), EGTA (1 mM), EDTA (2 mM), containing PMSF (1 mM), pepstatin (1 mM), leupeptin (2.2 μM), and benzamide chloride (0.5 mM)] on ice. The cell lysates were centrifuged (12,000*g*, 4 °C), and then the supernatants were used to estimate the protein concentration using the Bradford method. In the following, equal amounts of each protein (100 μg), the colorimetric caspase-3 substrate (AcDEVD-pNA, 5 μL of 2 mmol/l) and assay buffer [Nacl (100 mM), HEPES (50 mM), DTT (10 mM), CHAPS (0.1%), EDTA (0.1 mM), Glycerol (10%)] were mixed, and the reaction mixtures were incubated (at 37 °C in darkness for 3 h) and the absorbance was measured at 405 nm using a microplate reader^68^.

### Protein expression

Immunoblotting of protein expression is a reasonable analytical method for evaluating molecular biological activities. Here, to better explain OEO mechanisms, we investigated the expression of Bcl-2 (anti-apoptotic) and Bax (pro-apoptotic) proteins. Treated and control cells were lysed in an ice-cold lysis buffer [PIPES, KCl, mgcl2, DTT, EDTA, EGTA containing PMSF, antipain, leupeptin, and aprotinin]. The lysates were spun for 10 min and passed 10–15 times through an insulin needle to enable cell breaking-up and prepare the viscous lysate. All these processes were performed on ice. The lysates were centrifuged at 12,000*g* for 5 min at 4 °C. Finally, the concentration of supernatant proteins containing cytoplasmic proteins was quantified using the Bradford Assay. 20 μg of protein lysates from each sample were separated by SDS–PAGE and then transferred onto PVDF membranes. The membranes were blocked with 5% skim milk for 1 h before incubated with primary antibodies for 12 h. The following primary antibodies were anti-Bax (ab32503), and anti-Bcl2 (ab32124), and anti-beta actin (ab8226). The membranes were then carefully washed and kept in a species-matched HRP-conjugated secondary antibody for 3 h. The unbound antibody was washed off and removed. The bound antibodies were then detected by emerging the film^69^.

### ROS assay

The intracellular ROS was measured using DCFH-DA in A549 cells. The 3 × 106 A549 cells were exposed to OEO (½ IC_50_ and IC_50_) and positive control (IC_50_) for 12 h in 6-well plates containing 3 ml RPMI-1640. After harvesting and washing with PBS, the cells were stained with DCFH-DA (20 μM) and incubated in the dark for 15 min, and the intracellular fluorescence was detected using flow cytometry (FACSCalibur, BD Biosciences, San Jose, CA, USA) with excitation and emission settings of 485–495 nm and 525–530 nm, respectively^70^.

### Statistical analysis

All biological experiments were performed at least three times (with triplicates in each experiment). Representative results were depicted in this report, experimental data processing was carried out using Microsoft Excel (2013), and data were presented as means ± S.D. Analyses of different assays were performed using one-way ANOVA followed by Turkey’s post-test with assuming the normal distribution and homogeneity of variance in data. Additionally, at least some of our tests are triplicates and we know that for ANOVA, it is better to have more tests hence, we used another statistical test, the Kruskal–Wallis test which is a non-parametric method, and obtained results were in agreement with the previous results.

In the tables and figures, mean values with different letters within a column are significantly different by the Tukey test at (*p* < 0.05). Additionally, in the figures where *p < 0.05, **p < 0.01, ***p < 0.001 and ****p < 0.0001 to compare treated samples to control ones.

## Conclusion

Considering the findings, based on GC–MS analysis, thymol, carvacrol, *p*-cymene, and γ-terpinene were introduced as basic ingredients of OEO. In this study, OEO, thymol, and carvacrol exhibited in-vitro strong antioxidant capacity, although the effect of OEO was greater than others. Additionally, pretreatment of LPS-induced macrophages with OEO emulsion, thymol, and carvacrol led to a reduction in NO and ROS production and down-regulation of iNOS and NOX mRNA expression. In-silico molecular docking and Molecular Dynamics (MD) simulation exhibited that thymol and carvacrol but no *p*-cymene and γ-terpinene may establish proper bonds in iNOS active site and thereby inhibit this enzyme. However, they did not show any evidence for NOX inhibition indicating other ways to reduce NOX activity by these compounds. Our data also showed that *p*-cymene and γ-terpinene have little effect on oxidative scavenging. In addition, this study showed that OEO possesses appropriate selectivity between cancer and normal cells and can control the proliferation of A549 cancer cell lines. Additionally, apoptotic death and related mechanisms in OEO-treated A549 cells were detected by fluorescence microscopy, flow cytometry, colorimetric assay, and western blot analysis, and ultimately, apoptotic mechanisms were confirmed through induction of caspase-3 activity, an increase of Bax/Bcl2 ratio, and also ROS generation. In sum, although the OEO showed strong antioxidant properties in different conditions, it could cause apoptosis by increasing the level of ROS in cancer cells. Thus, our results provided new insight on the usage of OEO and main components, thymol, and carvacrol, into the development of novel antioxidant and anti-cancer agents. In other words, the structures of thymol and carvacrol could be used as templates for the design of new drugs in the future. However, the anti-cancer effects of OEO, thymol, and carvacrol and underlying mechanisms need more studies in the future.

## Supplementary Information

Supplementary Figures.
