# Supplementary material for: In-vitro, in-vivo, and in-silico assessment of radical scavenging and cytotoxic activities of Oliveria decumbens essential oil and its main components
Source: Sci Rep. 2021 Jul 12;11:14281. doi: 10.1038/s41598-021-93535-8 (PMC8275595; doi:10.1038/s41598-021-93535-8)
Supplement: Supplementary file 1 — Supplementary Figures. [file 41598_2021_93535_MOESM1_ESM.docx]

***In-vitro*, *in-vivo,* and *in-silico* assessment of radical scavenging and cytotoxic activities of *Oliveria decumbens* essential oil and its main components**

Tahereh Jamali^a,b^, Gholamreza Kavoosi^c^, Yousef Jamali^d^, Saeed Mortezazadeh^e^, Susan K. Ardestani^a^*

a. Institute of Biochemistry and Biophysics, University of Tehran, Tehran, Iran

b. Immunoregulation Research Center, Shahed University, Tehran, Iran

c. Institute of Biotechnology, Shiraz University, Shiraz, Iran

d. Biomathematics laboratory, Department of Applied Mathematics, School of Mathematical science, Tarbiat Modares University, Tehran, Iran

e. Department of Biophysics, Tarbiat Modares University, Tehran, Iran

* Corresponding author: Susan K. Ardestani Email: Ardestany@ut.ac.ir

**Gel raw figures:**


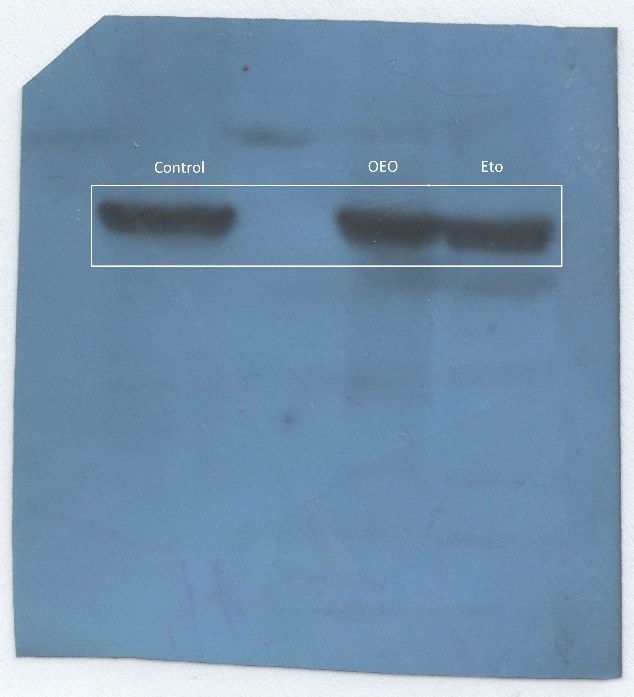


Figure S1: **β-actin**


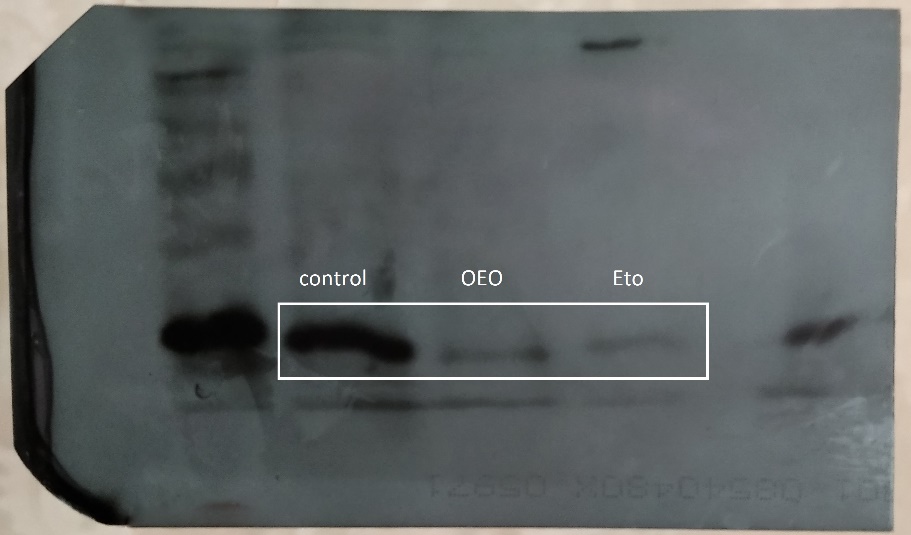


Figure S2: **Bcl-2**


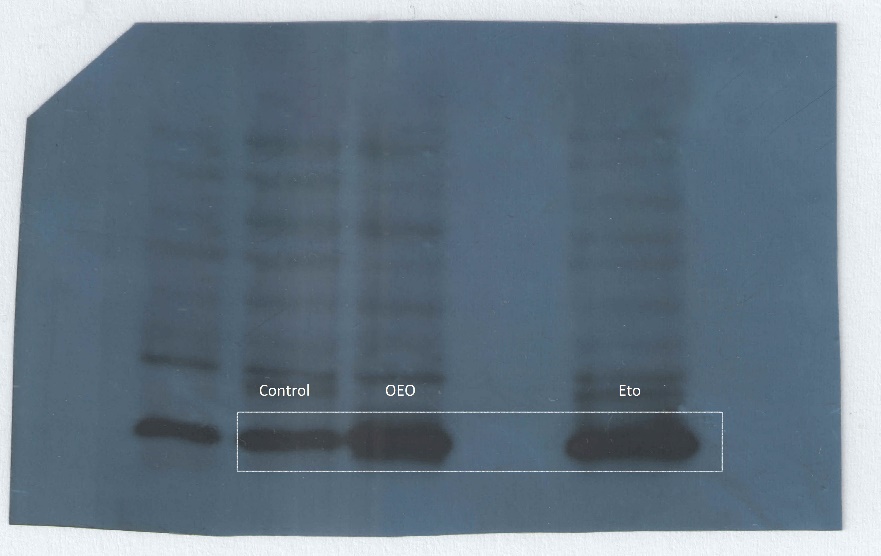


Figure S3: **Bax**
